# Some Thoughts on Modern Medicine1Presidential address at the 28th annual meeting of the Alumni Association, University of Buffalo, Medical Department.

**Published:** 1903-07

**Authors:** A. W. Bayliss

**Affiliations:** Buffalo, N. Y.; 592 Spring Street


### Some Thoughts on Modern Medicine.[1]

By A. W. BAYLISS, M. D., Buffalo, N. Y.

IT CERTAINLY gives me great pleasure, and I consider it a great honor, to be with you today as president of this body of 2,000 physicians. Although I am not, strictly speaking, one of your graduates, we are all like the graft upon the older trunk. We receive our strength from the same source, and the duties we perform are the same general duties which fall to all of us; for we must all belong to the same family, as it is a known fact that you cannot graft a plum or cherry upon an apple tree and make it grow and flourish, but how easy it is to make different members of the same species grow and flourish together, and in the same way I hope we will continue to do, as we have in the past. Even if we, of old Niagara, have no alumni association, we have found a stronger parent tree to which we are engrafted and from which we are receiving the same strength and assistance as if we were of the parent tree itself.

It is my intention to speak today of some of the modern remedies and helps in the practice of medicine. Practically, the greatest change in medicine that has taken place since I

1. Presidential address at the 28th annual meeting of the Alumni Association, University of Buffalo, Medical Department.

graduated is the serum therapy, which has entirely revolutionised the treatment of some diseases, and a great amount of work is continually being done to perfect this line of treatment. Also, the passing from antisepsis to asepsis in surgery and obstetrics is quite an important departure from a few years ago. Much might be said of the change in operative technic, but I will not speak of that, as I am not a surgeon.

In regard to preventive medicine, this seems to be the beginning of that epoch, for, during the past few years, much work has been done, and I think in the next few years much more will be done, as the work yet to be accomplished is a great one. When we see the terrible death-rate from consumption and realise that with proper care this disease can be prevented from spreading as it does, then we see the necessity for renewed efforts along this line. Not only America has awakened to this danger, but all the civilised nations are doing much to prevent the spread of this disease. This has been brought about by the thorough and scientific study of pathology and bacteriology. I think we, as physicians, should at all times lend our support to any means that will help to prevent the spread of this terrible disease. There are many other ills that much can be done to prevent; but none, I think, has the importance of tuberculosis. When we call to mind our feelings ten years ago upon every call to treat a case of diphtheria, how our hands were tied, as we might say, and compare the confidence with which we now approach the same class of patients, what a contrast, and what a sense of security and confidence we feel if called in time. Imagine if you can our sensations when we can call on our consumptive patients with the same confidence and expectations.

One of the newer assistants we have in medicine, or rather an old one brought into use again, is electricity. This something has been used for centuries by physicians for the cure of disease, but never until within a few years has it come into such intelligent use. This being an abstract power, not visible, our ancestors could only see the effect and know from it that they were dealing with something which had been a source of study for years, but which they were unable to comprehend or to apply with any certainty of action. Now, with improved instruments, and with increased knowledge in its application, we know conclusively that we have something which we can apply as an assistant and see its immediate effect. Formerly it was necessary to take for granted certain assertions made by the investigators, as the experiments made by them were too vague and tedious to be taken up by the busy practitioners; but since the discovery of the *x*-ray, we have evidence of the usefulness of this agent almost daily in our work. This is only one of the many ways of the daily use of this agent in medicine.

We must not think this factor is any different from others we have to deal with; that it can be used unintelligently or with carelessness. An expression we formerly heard is now seldom mentioned—namely, that "electricity will do no harm if it does no good." We have learned that harm can come from its use, for we occasionally find those who are electro-opposite, yet not so often as those whom we might call, for want of a more accurate term, electro-anemic. There is no way of determining who these patients are until we bring them into application with the electrical elements; neither can we formulate any rule unless at the same time we formulate many exceptions to it. This field is too wide to treat of all the uses to which electricity may be put, hence I will confine my further remarks to the *x*-ray and some of its uses.

Practically at no time has any means of cure come into such general use in so short a time as this, and yet this city was probably one of the slowest to adopt it. However, after it once took foothold, it became a popular electrical method. We have two means of exciting the vacuum tube—namely, the coil and the static machine, and they each have their advocates. I favor the induction coil, as it can be regulated with much greater ease than the static machine, which is one of the principal agencies of the use of the ray, both in radiotherapy and radiography; for we seldom have two cases that need the same current, and by being able to vary the strength of the current we can treat our patients as we wish, or intensify the tube if we wish to radiograph the deeper structures. Another indispensable part of the apparatus is a tube that can be regulated properly; for, unless we have this, several tubes of different penetrability become necessary in treating deep-seated tumors of the abdomen.

Then there are cases which must be acted upon differently, even if located upon the skin; in some cases we need the cauterising effect, while in others we desire simply the corrective change in the cell, which the long-continued use of the *x*-ray seems to produce; and these different features we cannot get unless we have some manner of varying the current strength. By changing the distance of the tube from the part treated, the number and to a certain extent penetrability of the ray may be varied, but as the rays diverge from the disc, by increasing the distance from the object, a very few rays are obtained, which are much weakened by their passage through this resistance of the ever varying conditions of the air.

In the future many of the diseases for which we are using this treatment will be found not benefited by it, and others that are not considered suitable for it will derive much benefit from its application. We can find a proper field for its use only by continued experiment. As we do not know how it acts we must yet work in the dark, but I think the time will soon come when it will be proven that this light acts upon the abnormal cell growth, by assisting nature to return the cell to its natural normal condition. This may be brought about in two ways. First, by the slow, weak and oft-repeated treatment which seems to assist nature in its effort to throw off disease; and, second, by the more radical treatment in which we get nearly the cauterising effect of the light, destroying the cell, as you may say, and allowing nature to replace it with a normal one. This latter method is much quicker than the former, and I think more dangerous, as nature is not always equal to the tax put upon it. So much for radiotherapy. Much more could be said, but I must hasten.

In regard to the use of the Finsen light, or ultra-violet rays, our neighbors across the water are doing a great deal with it, but the cases in which they use it are not plentiful here. The principal use they make of it is in the treatment of lupus, rodent ulcer and a few cases of epithelioma. Within the past year there have been many improvements and changes made in the Finsen light. Some are made to act directly from the arc, while others are used with a coil and condenser, taking all the different forms that the fancy of the manufacturer may lead him to. One that I have seen is an arc light in which the carbons are combined with iron, from which they claim to derive a more effective action, but the experiments are too recent to determine this question with certainty. As this light is of no use whatever, unless the parts to which it is applied are made bloodless, and the most practical way of obtaining this is by pressure, and as this treatment takes from a quarter to half an hour, it requires the undivided attention of the operator for that time, which is very tiresome, much more so than the use of the *x*-ray, and with very few exceptions we can do the same work with the *x*-ray as with the Finsen light.

In regard to the use of the $x$-ray for diagnostic purposes, it would not be easy for me to dispense with it. Our sense of sight is practically one of the acutest of the five. We can educate our sense of feeling to a high state of perfection, but never to be equal to the eye; when we can see upon the negative or upon the screen fractures never known to exist, then we may surely say this opens up a new field for study. So far there has been little done in radiography of the soft tissues, yet something has been accomplished and more will be done in the future with the assistance of the plate maker, who, we hope, will in time be able to make a plate more sensitive to these rays. Then we may expect to be able to see the soft tissues and diagnosticate with the eye that for which we must now use the sense of feeling and the knife.

In conclusion, permit me to again thank you for the honor conferred upon me and to express the hope to be able to meet with you in the future many times, and especially to see the continued success of the Alumni Association, which can only be brought about by the same united action which has characterised the past history of its meetings.

592 Spring Street.

